# Strategy for the Biosynthesis of Short Oligopeptides: Green and Sustainable Chemistry

**DOI:** 10.3390/biom9110733

**Published:** 2019-11-13

**Authors:** Tao Wang, Yu-Ran Zhang, Xiao-Huan Liu, Shun Ge, You-Shuang Zhu

**Affiliations:** School of Biological Science, Jining Medical University, Jining 272000, China; todrzhang666@163.com (Y.-R.Z.); Xhz_lxh@outlook.com (X.-H.L.); geshun@163.com (S.G.)

**Keywords:** short oligopeptides, biosynthesis, non-ribosomal peptide synthesis, ATP-grasp enzyme, β-lactam, cyclic dipeptides, metabolic engineering

## Abstract

Short oligopeptides are some of the most promising and functionally important amide bond-containing components, with widespread applications. Biosynthesis of these oligopeptides may potentially become the ultimate strategy because it has better cost efficiency and environmental-friendliness than conventional solid phase peptide synthesis and chemo-enzymatic synthesis. To successfully apply this strategy for the biosynthesis of structurally diverse amide bond-containing components, the identification and selection of specific biocatalysts is extremely important. Given that perspective, this review focuses on the current knowledge about the typical enzymes that might be potentially used for the synthesis of short oligopeptides. Moreover, novel enzymatic methods of producing desired peptides via metabolic engineering are highlighted. It is believed that this review will be helpful for technological innovation in the production of desired peptides.

## 1. Introduction

Short oligopeptides, especially l-α-dipeptides and their derivatives, are the simplest amide bond-containing components. However, they display various special and interesting biological activities, including taste-enhancing, antibacterial, nutritional, and anti-tumor activities [1] (Table 1). These activities are mainly due to the special structures of dipeptides. A dipeptide can be seen either as a derivative of an amino acid or as the dipeptide itself. As a derivative of an amino acid, the dipeptide and the parent amino acid usually show different physicochemical properties but the same biological effects, because dipeptides, such as Ala-Gln and Gly-Tyr, can be degraded into individual amino acids via specific proteases in organisms. In contrast, various dipeptides, such as aspartame and carnosine, have unique bioactivities that cannot be found in the parent amino acids. Compared with the numerous studies on the function, application, and preparation of proteins or amino acids, the research progress on dipeptides has been relatively slow, and only a few dipeptides, such as Ala-Gln and aspartame, are available. One of the major reasons is the lack of cost-effective and efficient manufacturing processes.

Various methods have been reported for the synthesis or formation of peptide bonds, such as chemical synthesis, chemo-enzymatic synthesis, and enzymatic synthesis (biosynthesis). The chemical synthesis of dipeptides usually includes four principal procedures [14]: (1) protection of functional groups, (2) activation of the free carboxy group, (3) formation of a peptide bond, and (4) removal of the protecting groups. With chemical synthesis, almost all designed dipeptides can be synthesized with appropriate protecting groups, and the yield is usually high. However, the disadvantages of chemical synthesis, such as the high cost, possibility of racemization, and lack of environmental-friendliness, are also very clear. The chemo-enzymatic synthesis of dipeptides results from the reverse reaction catalyzed by peptide bond-hydrolyzing enzymes (proteases or esterases). This method includes two distinct types of reaction processes: the thermodynamically controlled process or the kinetically controlled process. The former is carried out to drive the equilibrium toward peptide synthesis with necessary interventions, such as the precipitation of synthesized dipeptides or reaction with a large excess of substrates. The latter is dependent on an acylated serine or cysteine protease, which will then undergo a competitive deacylation process with water and the other amino acid. This method leads to temporary accumulation of the formed dipeptide. Compared with the chemical synthesis process, these two methods usually involve stricter stereoselectivity and milder conditions. However, they are usually influenced by many complicated factors such as severe hydrolytic side-reactions, the racemization-free preparation of activated peptide esters, and the limited availability of efficient peptide coupling and enzymes with high catalytic performance.

The synthesis of peptides by amide bond formation between specific (or partially protected) amino acid derivatives is, unfortunately, one of the most wasteful and least green chemical processes [15,16]. Due to the rapid development of DNA sequencing, tremendous progress has been made in the technologies of metagenomics, proteomics, and metabolomics, which lead to the identification of various enzymes that could be used to efficiently catalyze the synthesis of dipeptides. Given the great advantages of dipeptide biosynthesis, this review details strategies for dipeptide biosynthesis. Recent successful biosynthesis processes are also highlighted.

## 2. Biocatalysts Available for the Biosynthesis of Short Oligopeptides

### 2.1. Enzymes Used as Biocatalysts for the Biosynthesis of Dipeptides

**Figure 1:**
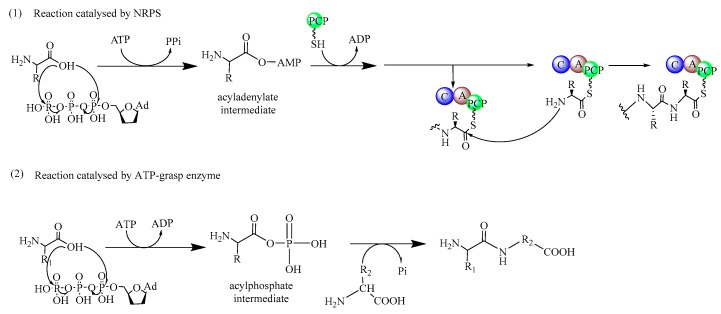
The reaction mechanism of two distinct types of biocatalysts for the synthesis of dipeptides. (**1**) Acyladenylate intermediates are formed in the process catalyzed by NRPSs. (**2**) Acylphosphate intermediates are formed in the process catalyzed by ATP-grasp enzymes.

Various enzymatic machineries that can catalyze the synthesis of dipeptides, such as the ribosome, non-ribosomal peptide synthetases (NRPSs), ATP(Adenosine triphophate)-grasp enzymes, and α-amino acid ester acyltransferases, have been found in nature [17]. These naturally occurring peptide-synthesizing enzymes seem to be ideal catalysts for dipeptide synthesis, even though they are diverse in their specificity and physiological function. However, one common feature is the requirement for ATP to catalyze the peptide bond-forming reaction. Based on the intermediates formed in the catalytic process, either aminoacyl-AMP (Adenosine monophosphate) or aminoacyl phosphate, these enzymes are generally divided into two categories [18]. The representatives of the former category are the ribosome, tRNA-dependent ligases, adenylate-forming amide ligases, and the NRPSs (Figure 1). The latter category usually contains a variety of enzymes, which usually shares a characteristic ATP-grasp motif in their amino acid sequence and are known as the ATP-grasp enzymes. This includes glutathione synthetase, d-alanine-d-alanine ligase, and other ATP-grasp enzymes.

#### 2.1.1. NRPSs

Non-ribosomal peptide synthesis is a universal and critical biochemical process catalyzed by NRPSs in bacteria and fungi, through which a wide array of therapeutically important peptides with highly diverse structures and bioactivities, including penicillin, bleomycin, and cyclosporine, are produced [19,20]. Biocatalysts capable of NRP synthesis could be divided into two groups (ATP-independent and ATP-dependent enzymes) [21] based on the difference in substrate activation [22,23]. In the ATP-dependent process, enzymes such as tRNA-dependent ligase can activate the substrate through aminoacyl-adenosine monophosphate. In the ATP-independent process, enzymes such as transacylase use aminoacyl phosphate.

**Figure 2:**
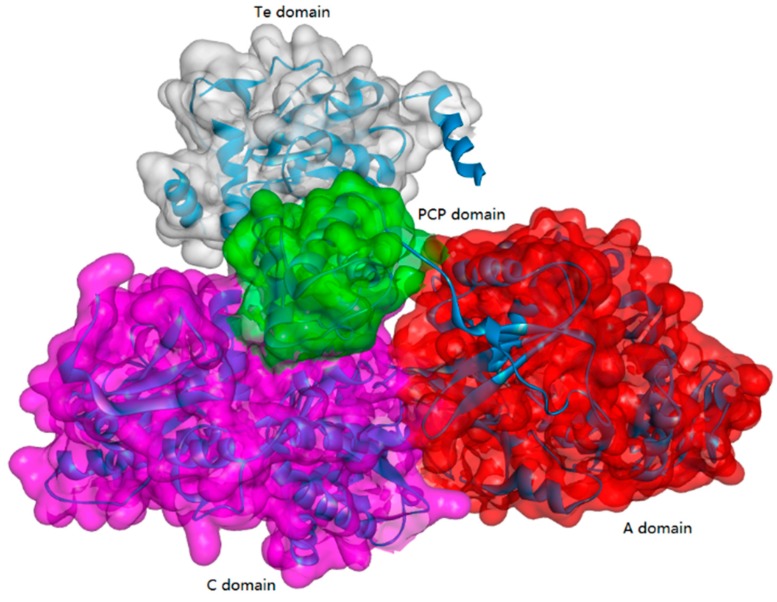
Structure of typical NRPS enzymes (PDB ID: 2VSQ, in this family, enzymes usually consist of the A domain, the C domain, the Te domain, and the PCP domain).

**Figure 3:**
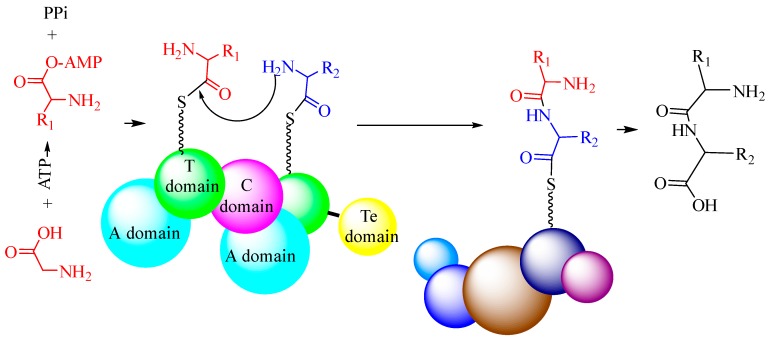
The process of amide bond formation catalyzed by NRPS enzymes.

NRPSs are large multi-functional proteins organized into different modules, where each consists of the catalytic domains responsible for incorporating one amino acid into the growing peptide product. A standard NRPS complex usually contains at least four enzymatic domains (Figure 2): the condensation domain (C-domain), the adenylation domain (A-domain), the thiolation domain (T-domain), and the thioesterase domain (Te-domain). The A-domain could activate a specific amino acid substrate as an aminoacyl adenylate. In the PCP domain, a thioester, aminoacyl-S-PCP, could be formed. The C-domain then catalyzes the amide bond formation by releasing a dipeptide. The Te domain is usually located at the end of the large proteins and is capable of terminating the biosynthesis of specific peptides. The process of NRPS-catalyzed peptide bond formation can be summarized as follows (Figure 3). First, the A-domain can identify the substrate amino acid and then activate it as an aminoacyl-AMP. Then, the activated amino acid is transferred to the 4′-phosphopantetheine moiety of the T-domain, accompanied with the release of adenosine monophosphate (AMP). Next, the peptide bond is formed in the adjacent condensation domain (C-domain). Lastly, the synthesized peptide is released from the NRPS complex by catalysis via the Te-domain. In addition, the PCP is a small 8 kDa domain that belongs to the non-overlapping members of a superfamily of carrier protein domains, which play many roles in acyl group transport.

Due to the superior catalytic activity and the unique reaction mechanism of NRPSs in dipeptide synthesis, modular manipulation of NRPS has been successfully applied to dipeptide synthesis. This approach is detailed in the following section.

#### 2.1.2. ATP-Grasp Enzymes

The ATP-grasp enzymes (or ATP-dependent carboxylate-amine ligases) activate carboxylic acids such as acylphosphate intermediates. ATP-dependent carboxylate-amine ligases are seen in many different biological systems, such as de novo purine biosynthesis, which is the assembly of the pentapeptide chain of peptidoglycan. Another biologically important example is RimK, which catalyzes the tandem addition of l-glutamic acids to the carboxyl terminus of ribosomal proteins.

**Figure 4:**
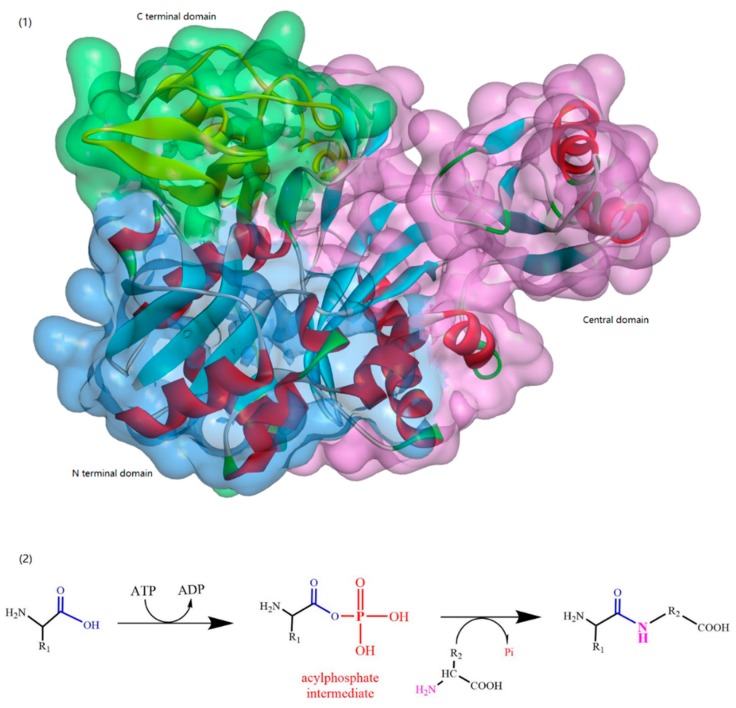
(**1**)A typical ATP-grasp enzyme: glycinamide ribonucleotide synthetase (PDB ID: 2IP4), and (**2**) the process of ATP-grasp enzyme-catalyzed peptide bond formation.

This family was the first of the amide bond-forming enzymes to be recognized and includes biotin carboxylase, d-Ala-d-Ala ligase (Ddl), and glutathione synthetase [24]. ATP-grasp enzymes are usually made up of three conserved domains (the N-terminal as well as central and C-terminal domains), which is a structure unique to this kind of enzyme (Figure 4). As their name implies, these enzymes usually have a nonclassical ATP binding fold comprising two α + β domains that “grasp” an ATP molecule between the central and C-terminal domains. Most ATP-grasp enzymes require an Mg^2+^ ion, which is coordinated by ATP in the active site.

##### l-Amino Acid Ligase (Lal)

**Figure 5:**
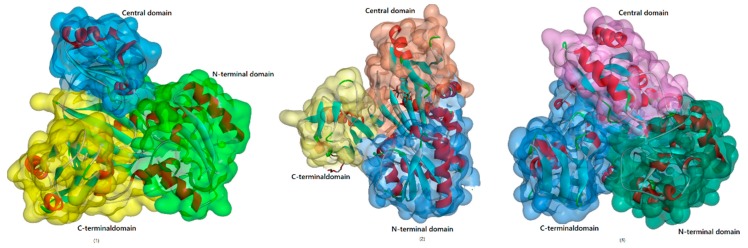
Typical l-amino acid ligases. (**1**) BL00235 (PDB ID: 3VOT); (**2**) RizA (PDB ID: 4WD3); (**3**) BacD (PDB ID: 3VMM). In this family, members consist of three conserved domains (the N-terminal, central-terminal, and C-terminal domains) and a nonclassical ATP binding fold comprising two α + β domains.

l-Amino acid ligase (Lal) is a special type of ATP-grasp enzyme that can catalyze only dipeptide synthesis from unprotected amino acids in an ATP-dependent manner (Figure 5). BacD (or YwfE, EC 6.3.2.28), which is identified from Bacillus subtilis in 2005 by Tabata et al. [25], was the first identified Lal. To date, several Lals have been identified and investigated, including RizA, Rsp1486a, BL00235, PSPPH 4299, plu1440, TabS, and FtyB (details shown in Table 2).

As shown in Table 2, almost all Lals identified to date show different substrate specificities, which leads to the production of different dipeptides. For example, the substrate specificity of BacD is restricted to smaller amino acids (e.g., l-Ala) at the N-terminal end of the dipeptide, whereas a wide range of hydrophobic amino acids (e.g., l-Phe) are recognized at the C-terminal end [26]. However, TabS can accept larger amino acids as its N-terminal substrate [27]. Plu1440 synthesizes dipeptides that contain l-asparagine at the N-terminus [28], and RSp1486a accepts bulkier amino acids as N-terminal substrates and less bulky amino acids as C-terminal substrates [29]. Based on these findings, it is reasonable to biosynthesize target dipeptides by modified Lals with improved substrate specificity [30]. This approach has provided novel methods for the production of useful dipeptides.

##### d-Alanine: d-Alanine Ligase

**Figure 6:**
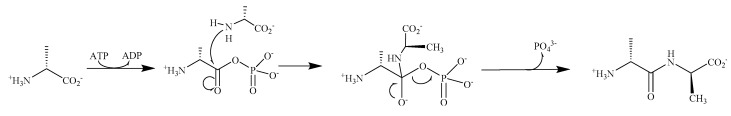
Proposed mechanism of Ddls.

Recently, it was reported that compared with l,l-dipeptides, d-amino acid-containing dipeptides have novel biological properties and are expected to be novel functional compounds for pharmaceuticals and food additives [35,36]. d-Alanine: d-alanine ligase (carboxylate-amine ligase, EC 6.3.2.4) is involved in the biosynthesis of the peptidoglycan component of the bacterial cell wall [37] and catalyzes the ATP-driven ligation of two d-alanine molecules, which results in the formation of d-alaninyl-d-alanine dipeptides (Figure 6). Given that Ddls do not have any homologue in humans, they have historically been considered promising targets for developing novel anti-bacterial components [38]. In addition to d-Ala-d-Ala, the formation of d-Ala-d-Ser dipeptides or d-Ala-d-Lac depsipeptides can also be catalyzed by Ddls [37].

##### The Poly-α-Glutamic Acid (αPGA) Synthetase RimK

**Figure 7:**
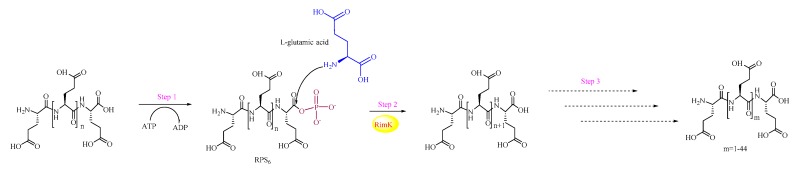
The reaction catalyzed is by poly-α-glutamic (αPGA) acid synthetase (RimK).

RimK is a member of the ATP-dependent carboxylate-amine/thiol ligase superfamily, which is reported to catalyze the modification of ribosomal protein S6 (RPS6) from Escherichia coli (E. coli) K-12 (Figure 7). In this biological process, Glu is added to the C-terminus of RPS6, which leads to the biosynthesis of RPS6-Glu, RPS6-Glu-Glu, RPS6-Glu-Glu-Glu, and RPS6-Glu-Glu-Glu-Glu.

In addition, Kino recently reported that RimK could catalyze the biosynthesis of αPGA from unprotected amino acids via ATP hydrolysis [39]. The results showed that the lengths of the resulting products changed with pH, and, at a pH of 9.0, a maximum 46-mer of Glu was obtained. RimK has strict substrate specificity for Glu. Therefore, it is possible to produce various biological Glu-containing products, such as dipeptides (e.g., l-glutamyl-l-glutamate and poly-glutamic acid) or tripeptides.

#### 2.1.3. α-Amino Acid Ester Acyltransferase

**Figure 8:**
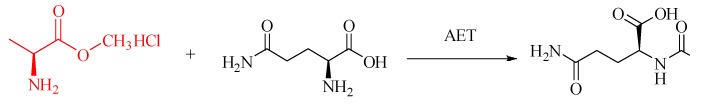
Reactions catalyzed by α-amino acid ester acyltransferase (AET).

Kenzo et al. [40] reported an efficient enzymatic method for producing oligopeptides from unprotected amino acids at a high yield. In this study, *Empedobacter brevis* ATCC 14234 was found to produce l-alanyl-l-glutamine (Ala-Gln) much more efficiently than previous methods. Furthermore, an enzyme catalyst (named carboxypeptidase Y) for the rapid production of Ala-Gln and other oligopeptides with unprotected substrates (l-alanine methyl ester hydrochloride, Gln, and more) was discovered in this strain and could be used to rapidly catalyze a reaction between l-alanine methyl ester hydrochloride (AlaOMe) and Gln to synthesize Ala-Gln. However, no additional detailed information, including the amino acid sequence, the coding gene sequence, or the 3D crystal structure, was reported in this study. Isao ABE et al. [41] first reported the cloning and expression of α-amino acid ester acyl transferases (AETs) from *Empedobacter brevis* ATCC14234 and *Sphingobacterium siyangensis* AJ2458. The proteins encoded are two similar polypeptides composed of 616 and 619 amino acid residues, respectively. Their amino acid sequences were 35% and 36% identical to that of the α-amino acid ester hydrolase from *Acetobacter pasteurianus*, respectively. AETs were believed to display dipeptidyl peptidase activity and transferase activity simultaneously. This enzyme was reported to use l-alanine methyl ester hydrochloride and Gln to synthesize Ala-Gln in a high yield (Figure 8) [42,43]. However, this enzyme also shows wide substrate specificity for both acyl donors and nucleophiles, which leads to the synthesis of not only dipeptides but also oligopeptides from different accepted substrates [40]. To date, there have been no studies on the 3D structure of α-amino acid ester acyltransferase and the detailed reaction mechanisms it catalyzes. These aspects should be explored further.

#### 2.1.4. Enzymes Used for β-Lactam Biosynthesis (β-Lactam Acylases)

**Figure 9:**
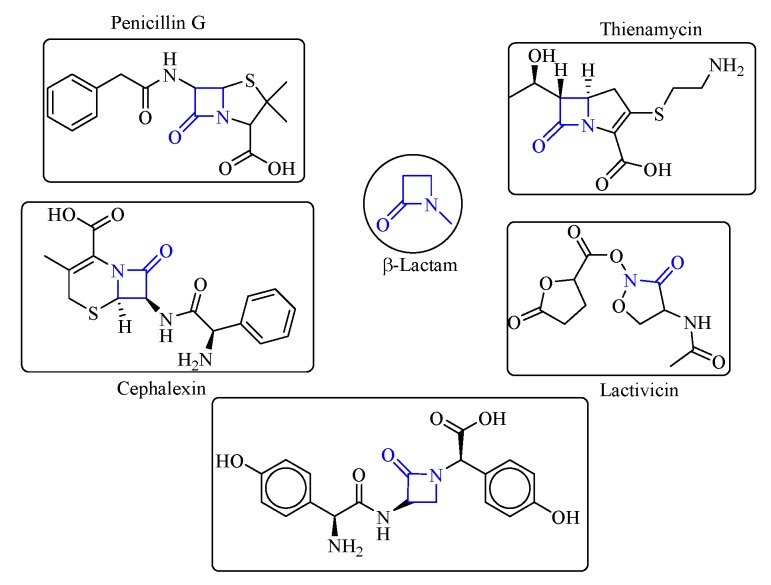
The chemical structures of β-lactam and several derivatives.

β-Lactam antibiotics are a large class of antibiotics containing a β-lactam ring in their chemical structure (Figure 9) [44], such as penicillin, cephalosporins, and thiamycins. β-Lactam antibiotics are among the most widely used clinical anti-infective agents and occupy an important position in the domestic pharmaceutical industry. The enzymatic synthesis of β-lactam antibiotics is more environmentally-friendly and economical than traditional chemical methods, with the advantages of mild reaction conditions, a clean and non-polluting nature, and good product quality. Therefore, this strategy has also been applied successfully in pilot-scale production in modern pharmaceutical enterprises. Traditionally, the β-lactam acylases are used for the hydrolytic processing of β-lactam antibiotics (e.g., penicillin G and cephalosporin C). However, some other acylases can also be used for the synthesis of semi-synthetic β-lactam antibiotics [45]. To date, several β-lactam acylases, including penicillin acylase (PA, EC 3.5.1.11), glutaryl acylase (GA, EC 3.5.1.93), and β-amino acid ester hydrolase (AEH, EC 3.1.1.43), have been widely investigated in the biosynthesis of β-lactam antibiotics. The biosynthesis of nocardicin is performed by an NRPS enzyme consisting of two mega-enzymes known as NocA and NocB [46].

Penicillin acylase is a well-known pharmaceutically important enzyme produced by various microorganisms. Based on the substrate specificity, PAs are further divided into penicillin G acylases (PGAs) [47] and penicillin V acylases (PVAs). The former preferentially hydrolyzes benzylpenicillin (pen G), and the latter preferentially hydrolyzes phenoxymethyl penicillin (pen V). These enzymes could be used on an industrial scale for producing the active pharmaceutical intermediate 6-aminopenicillanic acid (6-APA) by cleaving the side chain from natural penicillins. In addition, they could be used for the potential synthesis of newer semi-synthetic antibiotics by coupling new acyl groups to free β-lactam nuclei. On this basis, PAs hold great potential for application in the field of novel drug development. For example, these enzymes could be used directly to catalyze the ligation of novel synthetic fragments (novel side chains and β-lactam nuclei). Similarly, the enzyme engineering (e.g., directed evolution or rational design) of PAs could be performed for the catalytic synthesis of novel drugs. These developments will help to further expand and increase the potential of β-lactam antibiotics for future biopharmaceutical applications. In addition, PAs are employed in peptide synthesis and in the resolution of racemic mixtures [48]. Due to their enantioselectivity and promiscuity [49], PAs can also be used for producing achiral and chiral compounds for the preparation of synthons and bioactive pharmaceutical intermediates on a laboratory scale.

Although PAs have gained a unique position among the enzymes used by the pharmaceutical industry, they have serious drawbacks, such as the strong inhibitory effect of the produced phenyl acetic acid and instability at alkaline pH values. Given these considerations, α-amino acid ester hydrolases (AEHs, EC 3.1.1.43) are a promising alternative for the synthesis of α-amino-containing cephalosporins. Naturally, AEHs are capable of the semi-synthesis of β-lactam antibiotics containing an amino group, such as cephalexin, cefaclor, cefprozil, and cefadroxil [50,51]. Since variations in the side chain can alter the biochemical properties of a β-lactam antibiotic, semisynthetic antibiotics with novel side chains show promise in the development of novel drugs to cope with drug resistance. However, the presence of a hydroxyl group at the *p*-position of the phenylglycine side chain has been reported to cause a drastic decrease in specificity (Kcat/Km) compared to that of the analogue without this hydroxyl group. To address the issue of decreased activity toward components with a *p*-hydroxyl group, Ye et al. [52] explored the possibility of improving the substrate specificity of AEH toward para-hydroxyl cephalosporin synthesis by site-directed mutagenesis. The results showed that Arg87, Ser131, and Y175 play important roles in substrate recognition and the V131S mutant showed a 64% increase in the maximum accumulation of the cefatrizine product.

**Figure 10:**
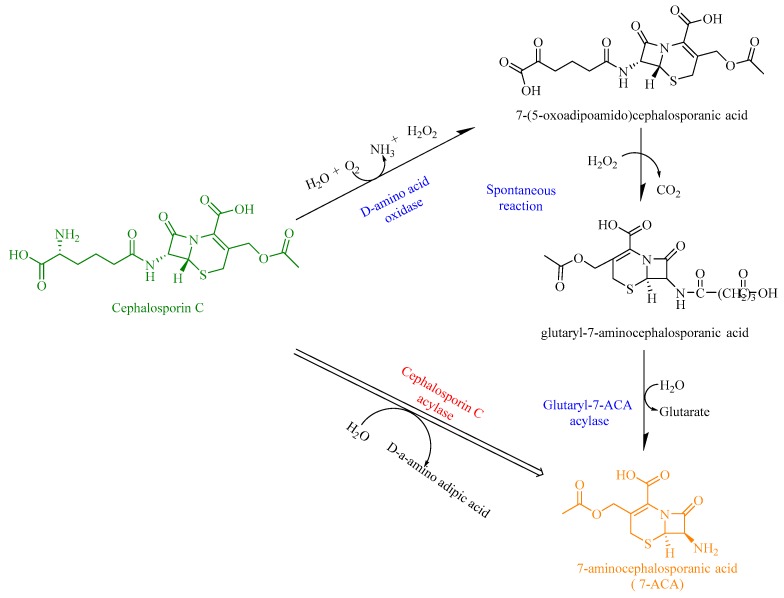
The reaction catalyzed by CephC acylase.

Glutaryl acylases are well-known industrial biocatalysts with wide substrate specificity (cephalosporin C (CPC) and/or glutaryl 7-aminocephalosporanic acid (GL-7ACA)) for producing 7-aminocephalosporanic acid (7-ACA) [53]. These enzymes have further been classified into five types (class I to class V) based on their gene structures (sequence conservation), substrate specificity, and enzyme properties [54]. All cephalosporin acylases are active toward GL-7ACA, but only members of classes I and III show appreciable activity toward cephalosporin C (CephC). Cephalosporin C acylases (CAs) [55] can specifically use CephC as their substrate to produce 7-ACA. 7-ACA is an important β-lactam nucleus for preparing many widely used semisynthetic β-lactam antibiotics [56]. In contrast, glutaryl-7-ACA acylases (GAs, EC 3.5.1.93) usually preferentially use GL-7ACA as their substrate. The most important application of GAs is the expensive and environmentally hazardous two-step enzymatic route for the synthesis of 7-ACA. As an excellent alternative, the single-step production of 7-ACA can be accomplished using CephC acylase (Figure 10) [57]. Unfortunately, natural CAs are usually efficient in the deacylation of GL-7ACA but are less active toward adipyl-7-ADCA and are barely able to hydrolyze CPC for the industrial production of 7-ACA [58].

#### 2.1.5. Cyanophycinases (CGPases)

**Figure 11:**
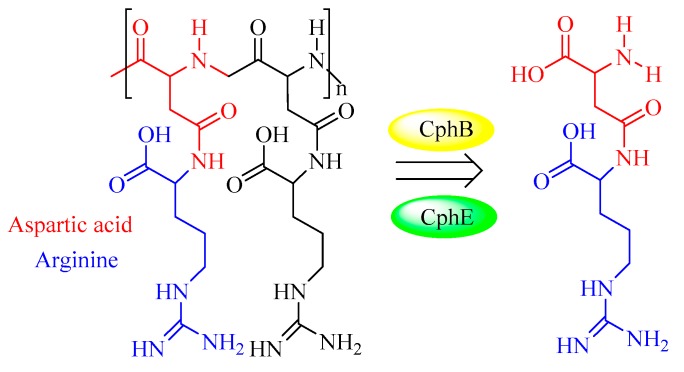
The reaction mechanism of cyanophycinases.

Cyanophycin granule polypeptide (CGP, or multi-l-arginyl-poly) is an intracellular storage polymer found in most cyanobacteria. Equimolar concentrations of arginine and aspartic acid are observed in the aspartic acid backbone, where the arginine moieties are linked to the β-carboxyl group of each aspartic acid through its α-amino group. In most genera of cyanobacteria, the cyanophycin synthetase gene (*cphA*) has been identified and verified for the synthesis of CGP. In contrast, the intracellular and extracellular degradation of CGP is catalyzed by cyanophycinases (CphB and CphE), which releases dipeptides (β-Asp-Arg, Table 3, Figure 11). In this respect, β-Asp-Arg can be efficiently synthesized via the simultaneous production of CGP and CGPase, which could be further applied in various fields requiring arginine (Arg) content in feed or food. However, the production and efficient isolation of CGP from various organisms have been successfully established in several recombinant strains, including *Escherichia coli* [59], *Nicotiana tabacum* [60], *Pseudomonas putida* [61], and *Pseudomonas alcaligenes* DIP1 [62]. Therefore, it is very feasible to produce dipeptides (e.g., β-Asp-Arg) via the metabolic engineering of suitable hosts [63] and chemo-enzymatic strategies [64]. For example, successful co-expression of CGP and CGPase in the *Nicotiana tabacum* plant was recently achieved [65]. A further study showed that it is possible to realize the goal of sufficient storage and efficient transport of arginine and β-Asp-Arg dipeptides in this synthetic model.

#### 2.1.6. Methods Used for the Biosynthesis of Cyclic Dipeptides

Cyclic dipeptides, or cyclodipeptides (CDPs), which are mainly produced by microorganisms as secondary metabolites, are the smallest cyclic peptides frequently found in nature and exhibit various noteworthy biological properties [71]. For example, cyclo(l-Phe-l-Pro), cyclo(l-Phe-trans-4-OH-l-Pro), clomycin, albonoursin, pulcherrimin, mycocyclosin, ambewelamides, and phenylahistin are several typical CDPs with potent antibacterial, antiviral, and immunosuppressive properties [72]. From a chemical structural perspective, CDPs are also called 2,5-diketopiperazines, and they are characterized by amide linkages formed to the two nitrogen atoms of a six-membered piperazine ring.

**Figure 12:**
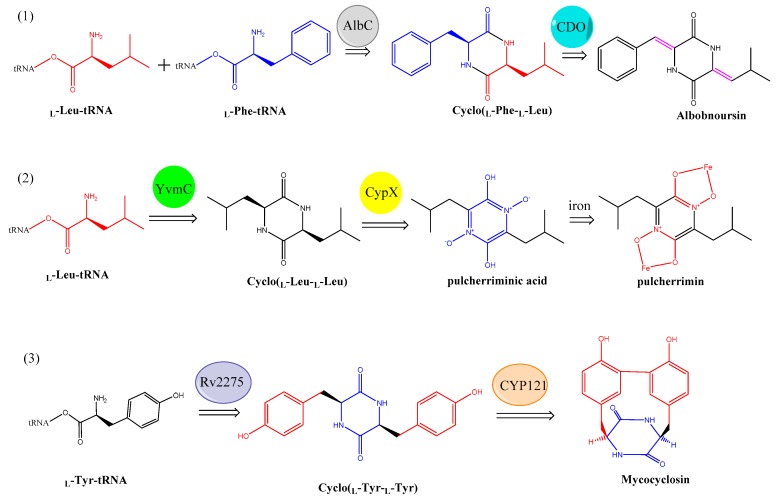
The reaction mechanism of typical cyclodipeptide synthases: (**1**) Albc, (**2**) YvmC, and (**3**) Rv2275.

In nature, the formation of the scaffold of CDPs is catalyzed by two unrelated biosynthetic enzyme families (Figure 12): either CDP synthases (CDPSs) [72] or non-ribosomal peptide synthetases (NRPSs) [73]. Subsequently, the resulting cyclodipeptides are usually further modified by tailoring enzymes, and the final CDPs are released [74,75,76].

**Figure 13:**
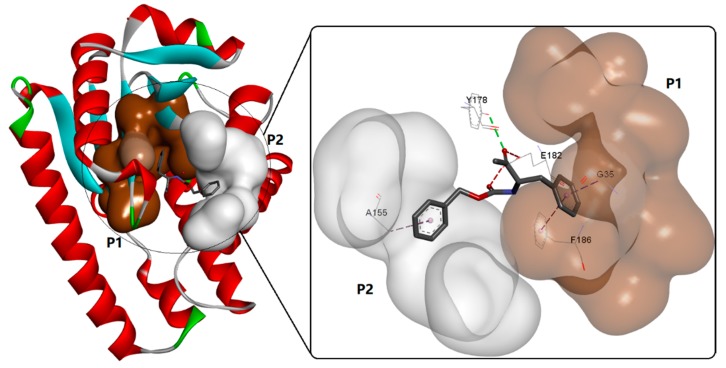
The typical cyclodipeptide synthase AlbC (PDB ID: 3OQV) and its substrate binding mode.

CDPSs, first defined in 2002, can connect primary metabolic pathways with secondary metabolic pathways by using aminoacyl-tRNAs as substrates to catalyze the formation of f diketopiperazines (DKPs) [75]. Based on the identity of three essential residues, CDPSs have been further divided into two subfamilies: NYH (e.g., AlbC and YvmC) and XYP (e.g., X40 and P203) [77]. Enzymes in the former subfamily are characterized by a typical structural architecture (Rossmann fold) used for substrate binding, which might lead to an exceptionally broad tRNA substrate specificity, producing various cyclodipeptides [78]. To date, the structures of six CDPSs can be retrieved from the RCSB PDB (https://www.rcsb.org/), including AlbC (PDB ID: 3OQV, Figure 13) from *Streptomyces noursei* [79], CDPS (PDB ID: 6EZ3) from *Staphylococcus haemolyticus* [80], CDPS (PDB ID: 4Q24) from *Streptomyces noursei* [81], CDPS from *Fluoribacter dumoffii* [80](PDB ID: 5OCD), CDPS from *Rickettsiella grylli* [80] (PDB ID: 5MLP), and CDPS from *Nocardia brasiliensis* [80] (PDB ID: 5MLQ). In addition, with the advent of next-generation sequencing, many more have been identified via BLAST (Basic Local Alignment Search Tool) searches and genome mining [82]. In addition to CDPSs, cyclic peptides could also be synthesized by NRPS modules [83]. In this study, BPSA prtein, which is a single NRPS module encoded by *bpsA*, was determined to be able to catalyze the synthesis of a blue pigment 5,5′-diamino-4,4′-dihydroxy-3,3′-diazadiphenoquinone-(2,2′).

Recently, the catalytic mechanism of CDPSs has been further investigated and reported to fit a ping-pong-type model [84] with two characteristic and conserved pockets known as P1 and P2 [77]. Catalysis starts with the binding of the first aa-tRNA (in the P1 pocket) and the subsequent transfer of its aminoacyl moiety to a conserved serine, which leads to the formation of an aminoacyl enzyme intermediate (in the P2 pocket). The aminoacyl moiety of a second aa-tRNA interacting with the pre-formed intermediate is then transferred to the aminoacyl enzyme, which leads to the formation of a dipeptidyl enzyme intermediate [81]. The final cyclodipeptide is released after intramolecular cyclization of the dipeptidyl moiety.

Considering the great potential of CDPSs in the biosynthesis of CDPs, it is believed that studies on the protein engineering of CDPSs will greatly facilitate the production of a variety of natural and unnatural bioactive cyclodipeptides [85,86].

#### 2.1.7. Biosynthesis of Imidazole-Related Dipeptides by Carnosine Synthase

**Figure 14:**
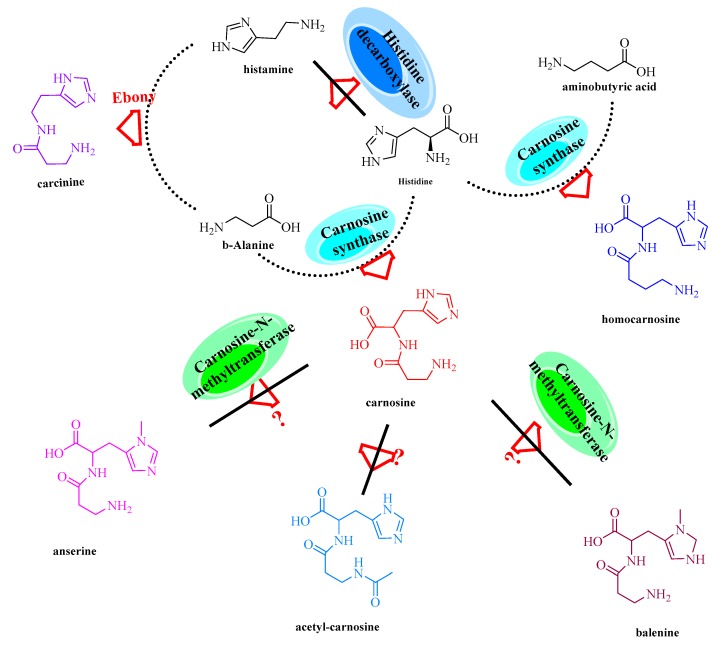
Biosynthesis of imidazole-related dipeptides by carnosine synthase.

Histidine dipeptides (or imidazole-related dipeptides), such as carnosine, anserine, ophidine, and homocarnosine, play a critical role in detoxifying cytotoxic reactive carbonyls and reversing protein glycation (Figure 14) [87]. Structurally, all of these enzymes contain a non-α-amino acid (β-alanine or γ-aminobutyric acid) at the N-terminus and an imidazole-related amino acid (histidine) at the C-terminus. They are widely distributed in the skeletal muscle, heart, and central nervous system of most vertebrates and some invertebrates.

**Figure 15:**
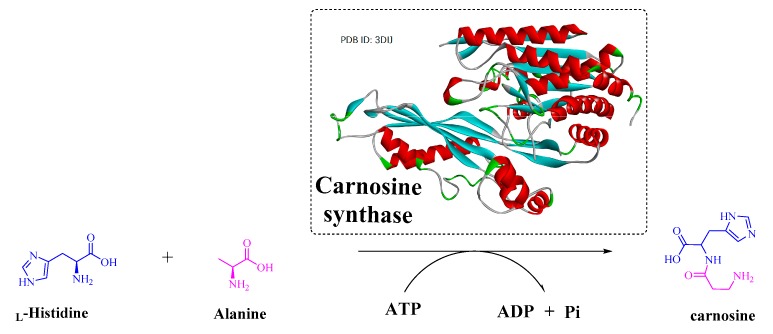
Crystal structure of carnosine synthase and the reaction catalyzed.

To date, three types of vertebrate enzymes have been identified for the biosynthesis of imidazole-related dipeptides: carnosine synthase (EC 6.3.2.11), carnosine *N*-methyltransferase (EC 2.1.1.22), and histidine *N*-acetyltransferase (EC 2.3.1.33). Histidine *N*-acetyltransferase is a type of *N*α-acetyl-histidine (NAH) synthesizing enzyme that can catalyze the biosynthesis of NAH with l-His and acetyl-CoA. Carnosine synthase is an ATP-grasp ligase that is one of the most important enzymes involved in the biosynthesis of anserine, homocarnosine, and carnosine (Figure 15) [88]. Similar to Lals, carnosine synthase is a catalytically promiscuous enzyme. Therefore, it can accept not only histidine but also lysine, ornithine, and arginine as C-terminal substrates to synthesize various dipeptides, such as β-Ala-Lys [89]. This promiscuity could also provide an efficient approach to modify the catalytic function of carnosine synthase to form novel natural or “unnatural” products.

#### 2.1.8. Proteases

Although proteases are primarily used for the hydrolysis of proteins and peptides, they can also be used to catalyze the kinetically or thermodynamically controlled formation of peptide bonds with unprotected substrate amino acids [90,91,92]. Thermodynamically controlled (or equilibrium-controlled) peptide synthesis can be achieved with all types of proteases. In contrast, kinetically controlled peptide synthesis is usually conducted with serine and cysteine proteases [93] because the specific triads (Ser-His-Asp and Cys-His-Asn) in these two enzymes can catalyze the transfer of an acyl donor to the acceptor (nucleophile) via the formed acyl–enzyme intermediate [94]. Therefore, the kinetically controlled method is more widely applied in biosynthesing oligopeptides, and various proteases, including papain, thermolysin, trypsin, α-chymotrypsin, and ficin, have been thoroughly explored [90,95].

In a study by Wei Qi et al. [96], papain, which is a commercially available and low-cost protease, was used successfully for the biosynthesis of *N*-(benzyloxycarbonyl)-alanyl-glutamine (*Z*-Ala-Gln) through a kinetically controlled strategy. The results showed that the dipeptide yield was 35.5%, and the apparent maximum reaction rate was determined to be 6.09 mmol/(L·min) under the optimized conditions. Wen-Yong Lou et al. proposed a novel method for the more efficient synthesis of dipeptides with the same biocatalyst (papain) in deep eutectic solvents [97]. In this study, papain was successfully immobilized onto magnetic nanocrystalline cellulose, and the obtained nano-biocatalyst (PA@MNCC) showed improved stability, enhanced solvent tolerance, and increased enzyme-substrate affinity. When this method was used for the synthesis of *Z*-Ala-Gln, the yield of the dipeptide in deep eutectic solvent was approximately 71.5%, which was the highest reported yield. This strategy is a competitive method for the synthesis of *Z*-Ala-Gln. Moreover, this study provided a promising carrier (magnetic nanocrystalline cellulose) that might be widely applied for enzyme immobilization.

## 3. Emerging Approaches for the Efficient Production of Short Oligopeptides: Rational Protein Engineering and Strain Development

### 3.1. Dipeptide Formation by Rational Engineering of NRPSs

NRPSs are modular ‘mega-enzymes’ that can catalyze the assembly of many smaller units, which produces various bioactive molecules. NRPSs can synthesize and assemble peptides in-line from amino acid monomers, which are first activated by the A domains and then loaded onto the adjacent carrier domains. Lastly, the formation of peptide bonds and transfer of the growing chain are catalyzed by the C domains. Because each module of NRPSs performs specific reactions, such as substrate activation, modification, and condensation, the rational arrangement of these specific modules (domain assembly and module fusion) for the design of novel engineered NRPSs to produce interesting products is very promising [98,99].

**Figure 16:**
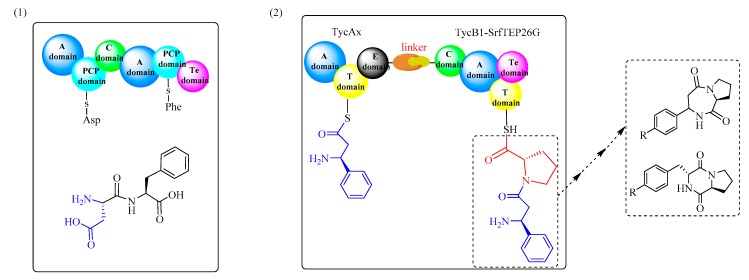
Dipeptide formation by the rational engineering of NRPS. (**1**) Rational fusion of the Asp and Phe activating modules and condensation domains, and (**2**) the biosynthesis of Asp-Phe catalyzed by the rationally constructed enzyme.

In a pioneering study by Marahiel et al. (Figure 16) [98,100], different Asp-Phe synthetases were designed and constructed through fusion of the Asp and Phe activating modules and condensation domains. The product formation assay showed that two different forms of Asp-Phe were successfully bio-synthesized (α-Asp-Phe and β-Asp-Phe), while enzyme III [A-PCP]SrfB2-[C-A]TycB2-[PCP-Te]TycC6 showed the best catalytic activity (Kcat = 0.7 min^−1^, α:β = 100:0). The turnover rates (ranging from 0.01–0.7 min^−1^) and the purity of α-Asp-Phe (75–100% of the overall product) indicate that the rational engineering of NRPSs shows great potential for the design and efficient production of novel dipeptides. However, it should be noted that the different fusion sites might play a critical role in the resulting catalytic activities of the fused catalysts.

Due to the chemical diversity covered by non-ribosomal peptides, rational modification of their backbones represents a promising strategy for the development of novel products with specific properties [101]. Therefore, determining how to produce the designed component via catalysis by an efficient biocatalyst might display great potential. From this perspective, engineering and reprogramming modular NRPSs to obtain novel catalysts with designed activities would make perfect sense. GrsA/GrsB1 is a truncated dipeptide synthetase excised from the gramicidin S NRPS [102], which can catalyze the biosynthesis of the d-Phe-l-Pro diketopiperazine. Based on this finding, Donald et al. [103] introduced a single W239S mutation in the phenylalanine-specific NRPS A-domain to enlarge the binding pocket. This modification greatly improved the activation process of unnatural aromatic amino acids functionalized with azide and alkyne groups. The results showed that the substrate specificity was increased by 10^5^-fold (for *p*-azido-l-Phe, Kcat/K_M_ = 9000 (25 for WT)) without appreciable loss of catalytic efficiency.

### 3.2. Engineering Modifications of Cephalosporin Acylase

Considering the nature of the similar chemical structures of glutaryl-7-ACA, adipyl-7-ADCA, and cephalosporin C, attempts have been made to create mutants of cephalosporin acylases with improved activities toward adipyl-7-ADCA and cephalosporin C. From this perspective, engineering modifications of cephalosporin acylase would be a feasible strategy to achieve this goal, and significant progress has been made in addressing the concerns of low substrate specificity, substrate inhibition, and product inhibition encountered in practice [104,105].

The study by Wim J. Quax [58,106] included a comprehensive mutational analysis of N266 and F375. The resulting mutations showed a broad spectrum of affinities and activities, which suggests the flexibility of the glutaryl acylase from Pseudomonas SY-77 at these positions. Moreover, the SY-77^N266Q^, SY-77^N266H^, and SY-77^N266M^ mutants also showed a modest improvement in cephalosporin C hydrolysis. In a study carried out by Zhanglin Lin et al. [107], a positive mutation, H57βA/H70βY, of the CPC acylase acyII from *Pseudomonas* SE8 with no substrate inhibition was obtained via two rounds of combinatorial active site saturation testing. Further study with a quick pH indicator assay designed for real-time monitoring and screening libraries of site-directed saturation mutations led to the discovery of a new mutation, H57βA/H70βY/I176βN, which showed a Kcat 3.26-fold when compared to the wild type. In this study, it was suggested that a larger binding pocket might better accommodate CPC as the optimal substrate. However, the reason that this mutant abrogates substrate inhibition remains unclear.

In addition to traditional molecular biology methods (such as random mutagenesis methods and directed evolution), rational protein design is a promising strategy in current enzyme engineering to improve enzymatic properties. In this field, several important studies carried out by Yu-shan Zhu et al. have demonstrated the importance of computational protein design [108,109,110,111,112]. In one study [111], molecular dynamics (MD) simulations and molecular docking were applied to investigate the dynamic features of active site-transition state complex structures of cephalosporin acylase to potentially avoid an excess of false positives produced by high-throughput screening. Through this approach, the limiting step and well-maintained geometrical constraints in the hydrolysis reaction of cephalosporin C were determined and revealed, which could be further used to improve the activity of cephalosporin C acylase. In other studies [109,110], computational protein design strategies were successfully used for enzyme engineering to increase catalytic activities (thermostability or activity). The cephalosporin C acylase from the Pseudomonas strain N176 was reconstructed and analyzed via the PROtein Design Algorithmic (PRODA) package [113]. Through this method, rational protein design for the improvement of stability and activity could be achieved simultaneously by analyzing the functions of the hydrophobic core regions and the regions surrounding the active sites. This study [110] revealed that the instability caused by introduced mutations (V68βA) at the active site could be reversed by repacking the nearby hydrophobic core regions (L154βF and L180βF). One study [112] achieved the computational redesign of native penicillin acylase active sites for the condensation reaction between d-dihydrophenylglycine methyl ester (DHME) and 7-ADCA, which produces cephradine in fully aqueous medium. The great advantage of this method might be the development of a scoring function based on discounted folding energy instead of the single binding energy or the overall folding energy (∆G^fold^). The results showed not only that the positive mutant (M142αF/F24βA/S67βA) displayed high substrate specificity but also that the catalytic activity was simultaneously increased by more than 10-fold. It is believed that this strategy provides a highly efficient and green approach to enzyme engineering to create novel biocatalysts for transforming a wide variety of substrates—both natural and unnatural compounds.

It is known that the protein structure determines the function and the structural, chemical, and physical factors that play important roles in catalytic activity and inevitably affect substrate specificity or stability [114]. Therefore, computational protein design could provide a promising platform for the design of novel industrial biocatalysts and for the study of protein structure and function, which has also become a leading field in the biophysical sciences [115,116,117].

### 3.3. Metabolic Engineering of Microorganisms for the Biosynthesis of Desirable Dipeptides

Ala-Gln is a very important compound from both the clinical and nutritional perspectives [118] and is the most suitable Gln-containing vector for the supply of l-glutamine (Gln). In addition to chemical synthesis and chemo-enzymatic synthesis, the metabolic engineering of *E. coli* for the biosynthesis of Ala-Gln has proven to be a promising strategy. Yoshinori Hirao et al. [42], Wenjie Yuan et al. [43], and Kino et al. [26] used α-amino acid ester acyltransferase and l-amino acid ligase as biocatalysts for the bio-catalysis of Ala-Gln. Wenjie Yuan engineered *E. coli* Origami 2 to produce Ala-Gln by overexpressing α-amino acid ester acyltransferase with the pET-29a(+) plasmid under the control of the T7 promoter. The engineered host could use l-alanine methyl ester hydrochloride (AlaOMe) and l-glutamine (Gln) as the substrates to synthesize Ala-Gln. The maximum molar yield and productivity were determined to be 94.7% and 1.89 g·(L·min)^−1^, respectively. Moreover, the high SsAet activity of α-amino acid ester acyltransferase maintained during the repeated cycle experiments could guarantee a high Ala-Gln yield.

An l-amino acid ligase, BL00235, was used and engineered for the selective synthesis of the salt taste enhancer Met-Gly [30]. Via site-directed mutagenesis of the P85 residue, the resulting P85F and P85Y mutants achieved selective Met-Gly synthesis without the synthesis of Met-Met. It was found that the key residues in the binding pockets (e.g., P85 of BL00235) play a critical role in substrate reorganization similar to that of BacD (Trp332). Therefore, rational modification of these sites would alter the substrate binding pockets, which leads to a restricted cavity for substrate binding. However, as seen from the abovementioned studies, a foreseeable result is that the obtained mutants showed lower yields but higher substrate specificities than the wild type. In our view, this difference arises because the catalytic performance of a specific catalyst is affected by various factors. Therefore, the simultaneous enhancement of multiple catalytic factors of L-amino acid ligase would be a feasible alternative for further studies.

## 4. Conclusions

Due to their specific structures and functionality, polypeptides often show remarkable chemical and biological properties. Therefore, they have been widely employed in various fields, such as biomedicine. Compared with conventional solid-phase peptide synthesis, the widely used chemo-enzymatic synthesis might be advantageous, especially for the biosynthesis of dipeptides or tripeptides, due to its environmental-friendliness and increased yields. However, critical challenges might be posed by the lack of insight into the detailed enzymatic mechanisms and difficulties in determining the optical reaction conditions.

In addition, the fermentative production of oligopeptides might potentially become the ultimate strategy. It is likely the most cost-efficient and environmentally-friendly approach. Thus, different types of key biocatalysts would be first used and engineered. Furthermore, the intracellular metabolic pathways of specific hosts would also be modified to redirect the metabolic flow of the substrate amino acids in order to suppress undesired pathways. As discussed above, the enzymes used (e.g., Lals) usually show broad substrate specificity. Therefore, the resulting spectrum of possible products would greatly affect the practical biosynthesis of oligopeptides. Protein engineering through directed evolution, rational design, and structure-based site-directed mutagenesis would help improve both the substrate specificity profiles and catalytic performance. Moreover, the use of engineered biocatalysts with improved catalytic performance would undoubtedly expand the scope of fermentative production of oligopeptides.

## Figures and Tables

**Table 1:** The dipeptides with interesting biological activities.

Function	Chemical Compound	Reference
Parenteral nutrition	Gly-Tyr	[2]
	Ala-Gln	[3]
Taste-enhancing	Sweetener: Aspartame	[4]
	Salt substance: Pro-Gly	[5]
Cytosolic buffering	Carnosine	[6]
Ophthalmic drug	*N*-Acetyl carnosine	[7]
Analgesic	Kyotorphin (Arg-Tyr)	[8]
Anti-tumor	Lys-Glu	[9]
Neuroprotective	Leu-Ile	[10]
Anti-bacterial	Bacilysin/Chlorotetaine	[11]
	rhizocticin	[12]
	tabtoxin	[13]

**Table 2:** Typical Lals and their functions.

Enzyme	Components Catalyzed		Availability of the Crystal Structure	Source	Ref.
	Natural Product	Unnatural Product			
BacD	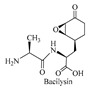	Ala-Gln	+, PDB ID: 3VMM	*Bacillus subtilis*	[25]
RizA	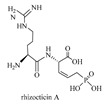	/	+, PDB ID: 4WD3	*Bacillus subtilis* NBRC 3134	[31]
PSPPH 4299	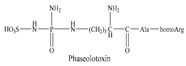	Ala-HomoArg, Ala-Arg Ala-Lys Ala-His Ala-Gln Ala-Asn Ala-Met Ala-Phe Ala-Trp	+, PDB ID: 3VMM	*Pseudomonas syringae* pv. *phaseolicola* 1448A	[32]
TabS	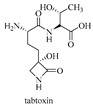	Arg-Phe Gln-Trp Leu-Ser Glu-Thr Leu-Ile Pro-Gly	-	*Pseudomonas syringae* NBRC 14081	[26]
FtyB	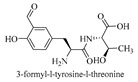	/	-	*Pseudoalteromonas tunicata* D2	[33]
Rsp1486a	Phe-Cys, His-Ala, His-Val, His-Gly	/	-	*Ralstonia solanacearum* JCM 10486	[28]
BL00235	Met-Gly, Met-Met	/	+, PDB ID: 3VOT	*Bacillus licheniformis* NBRC 12200	[29,34]
plu1440	Asn-Gly, Asn-Ala, Asn-Cys, Asn-Gln	/	-	*Photorhabdus luminescens* subsp. *laumondii* TT01	[27]

**Table 3:** Enzymes used for the degradation of CGP.

	Enzyme	Source	Product	Reference
Intracellular	Cyanophycinase (CphB)	*Anabaena cylindrica*	β-Asp-Arg	[66]
Extracellular	CphE_Pa_	*Pseudomonas anguilliseptica* BI	β-Asp-Arg	[67]
	CphE_Bm_	*Bacillus megaterium* BAC19	Small molecules	[68]
	CphE	*Sedimentibacter hongkongensis* KI	β-Asp-Arg, β-Asp-Lys	[69]
	CphE	*Pseudomonas alcaligenes* DIP1	Ethanol, acetic acid, succinic acid	[70]
